# Higher Levels of Osteoprotegerin and Immune Activation/Immunosenescence Markers Are Correlated with Concomitant Bone and Endovascular Damage in HIV-Suppressed Patients

**DOI:** 10.1371/journal.pone.0149601

**Published:** 2016-02-25

**Authors:** Alessandra D’Abramo, Maria Antonella Zingaropoli, Alessandra Oliva, Claudia D’Agostino, Samir Al Moghazi, Giulia De Luca, Marco Iannetta, Gabriella d’Ettorre, Maria Rosa Ciardi, Claudio Maria Mastroianni, Vincenzo Vullo

**Affiliations:** Department of Public Health and Infectious Diseases, Istituto Pasteur-Fondazione Cenci Bolognetti, “Sapienza” University of Rome, Rome, Italy; University of Cape Town, SOUTH AFRICA

## Abstract

HIV-infected patients appear to have a significantly greater risk of non-AIDS comorbidities such as osteoporosis and atherosclerosis. Subjects with osteoporosis are at a higher risk of developing cardiovascular disease than those with normal bone mass, therefore a possible relation between these two conditions can be hypothesized. In the setting of HIV infection, several factors might contribute to bone disease and endothelial dysfunction. The aim of our study was to evaluate the relationship between bone and cardiovascular disease and to investigate the role of traditional factors, T-cell phenotype and osteoprotegerin in HIV positive subjects on effective antiretroviral therapy. We included 94 HIV positive subjects on antiretroviral therapy with virological suppression and 41 healthy subjects matched for age and gender as a control group. Carotid-Intima Media Thickness (c-IMT) and bone mineral density (BMD) were performed by ultrasound and DEXA, respectively. CD4^+^/CD8^+^ T-cell activation, senescence and osteoprotegerin plasma levels were measured by flow-cytometry and ELISA, respectively. Among HIV positive patients, 56.4% had osteopenia/osteoporosis and 45.7% had pathological c-IMT (>0.9mm). Subjects with pathological c-IMT and BMD exhibited higher CD4^+^ and CD8^+^ activated, CD8^+^ senescent and osteoprotegerin than subjects with normal c-IMT and BMD. HIV positive subjects with osteopenia/osteoporosis had higher c-IMT than subjects with normal BMD, and linear regression analysis showed a negative correlation between BMD and c-IMT. Several factors are implicated in the pathogenesis of non-AIDS comorbidities in HIV positive patients. Osteoprotegerin together with inflammation and immunosenescence in HIV positive patients could affect bone and vascular system and could be considered as a possible common link between these two diseases.

## Introduction

With increasing life expectancy, HIV-infected patients appear to have a significantly greater risk of non-AIDS comorbidities such as osteoporosis and atherosclerosis, important causes of morbidity and mortality [1–3]. These two conditions have always been considered as unrelated and their coexistence was attributed exclusively to age-related processes. Recently, a connection between these two pathological conditions has been considered possible as they share genetic and pathophysiological risk factors [4–6]. Recent studies have shown that subjects with osteoporosis are at higher risk of developing cardiovascular disease than those with normal bone mass; furthermore, studies in animal models have shown that vascular calcification have mechanisms similar to those used in normal bone calcification [7]. In the setting of HIV infection, chronic inflammation, immune activation, immunosenescence and the consequent increased levels of pro-inflammatory cytokines might contribute to bone disease and endothelial activation/dysfunction [8–13]. The role of inflammation in the development of non-AIDS related chronic complication has been extensively studied and several markers such as TNF-*α*, IL-1 and IL-6 have been shown to reliably indicate organ damage [14–17]. Furthermore, new soluble markers including the RANK (Receptor activator of nuclear factor kappa-B)/RANKL (Receptor activator of nuclear factor kappa-B ligand)/OPG (Osteoprotegerin) axis, a member of the TNF superfamily, have emerged as a possible link between osteoporosis and cardiovascular disease. Mostly implicated in bone remodeling, the RANK/RANKL/OPG axis is involved in immune and vascular system. In fact, RANKL, expressed by osteoblast cells and their precursor, activates its receptor (RANK), expressed by osteoclast cells and their precursor, thus promoting osteoclast formation, activation, and prolonging osteoclast survival. The effects of RANKL are blocked by the secretory glycoprotein OPG, which acts as a decoy receptor for RANKL [18–20]. Perturbations of the RANKL/OPG ratio are critical in the pathogenesis of bone disease and have also been associated with changes in macrophages, dendritic cells and lymphocytes, which are involved in systemic inflammation, osteoclastogenesis and atherogenesis [21–23].

The aim of our study was to evaluate the relationship between bone and cardiovascular disease and to investigate the role of traditional factors, T-cell phenotype and OPG in HIV positive subjects on effective antiretroviral therapy (ART).

## Materials and Methods

### Ethics statement

The study protocol designed according to the Helsinki Declaration II was approved by the ethics committee of Azienda Policlinico Umberto I of Rome. All the patients gave written informed consent to participate.

### Patients

We recruited 94 HIV-infected subjects who had been on ART for 48 weeks with undetectable viremia (<37 copies/ml) at the Department of Public Health and Infectious Diseases of “Sapienza” University of Rome. As a control group, 41 HIV negative individuals matched for age and gender were included. For each patient, we collected medical and family history, lifestyle, smoking *status*, current and past ART, HIV-RNA zenith and nadir CD4^+^ cell count. Current T lymphocytes CD4^+^ and CD8^+^ cell count was determined by flow cytometric analysis (MACSQuant Analyser Miltenyi Biotec, Germany) and HIV-1 RNA plasma levels were detected by a quantitative reverse polymerase chain reaction (Amplicor HIV monitor; Roche Diagnostic System, Branchburg, NJ version1.5, l.o.d. 37 copies/ml). Calcium, vitamin D, triglycerides, total cholesterol, high-density lipoprotein cholesterol (HDL) and low-density lipoprotein cholesterol (LDL) were measured in blood samples. Systolic and diastolic Blood Pressure (SBP and DBP) (mmHg), Body mass index (BMI) (kg/m^2^) and Framingham score (%) were calculated and recorded for each individual. HIV positive subjects were stratified according to the presence of two comorbidities (concomitant bone and endovascular damage, Group A) or only one (bone or endovascular damage, Group B) or no comorbidity (Group C). Inclusion criteria were: age>18 years old; HIV positive subjects on effective ART (HIVRNA<37 cp/ml) since 48 weeks, HIV positive subjects with low cardiovascular disease risk (Framinghamm risk score<10%,) and CMV positive serostatus.

Exclusion criteria were age <18 years, previous virological failure, Framingham score >10%, CMV negative subjects, recent AIDS-defining illness, co-infection with hepatitis virus and presence of other comorbidities (metabolic syndrome, diabetes mellitus, arterial hypertension, kidney disease and hormonal dysfunction).

### Sample analyses

OPG levels were measured by ELISA kits (Biomedica Gruppe,Vienna, Austria). The detection limit of assay was 0.12 pmol/l. Lymphocyte surface phenotypes were evaluated by flow cytometry using fresh peripheral blood. For the activation and the senescence analysis of T cells CD4^+^ and CD8^+^, the following fluorochrome-labeled antibodies were used: Pacific Blue-CD3 (BioLegend, 500 uL), FITC-CD28 (BioLegend, 2 mL), PE-CD57 (BioLegend, 2 mL), PerCp/Cy5.5-HLA DR (BioLegend, 500 uL), Pe/Cy7-CD8 (BioLegend, 2 mL), CD38-APC (BioLegend, 2 mL), APC/Cy7-CD4 (BioLegend, 2 mL). The flow cytometer was calibrated using MACSQuantTM Calibration Beads and for the automatic compensation marked beads (BDTM CompBeads Anti -Mouse Ig, k) and unmarked beads (BDTM CompBeads Negative Control, FBS) were used. For the lysis of red blood cells FACS Lysing Solution (BD, Lysing Solution) was used. The flow cytometer used for the analysis was the MACSQuant Analyser with 8 channels and the data were analyzed by using the FlowJo V10 Software. Before each acquisition, both calibration and automatic compensation were performed. Immune activation was defined as HLADR^+^ CD38^+^ whereas immunosenescence was defined as CD28^-^CD57^+^ [24–25].

### Carotid intima media thickness measurement (c-IMT)

c-IMT measurement was obtained for each patient using a B-mode ultrasound recording with a 7- to 14-MZ array probe (ESAOTE-technology). Patients had to lie in a supine position in a dark room with a slight hyperextension and turning the neck to the opposite side. The common carotid, the bifurcation and at least the first 2 cm of the internal carotid were examined on the long and short axes. In addition, 3 measurements were made at the far and near walls of each internal carotid and specifically at the carotid bifurcation, the bulb and 1 cm after the bifurcation. The mean value (expressed as mm) of the 3 measurements taken at each site of the internal carotid (left and right) was calculated for each patient and used as the final measurement of internal c-IMT. According to published population studies, we defined normal c-IMT as IMT<0.9 mm and pathological c-IMT as IMT>0.9 mm. All the measurements of c-IMT were performed by a single operator to avoid inter-operator differences [26].

### Dual-energy X-ray absorptiometry (DEXA)

Bone densitometry using a DEXA device (Hologic QDR 2000 plus, Bedford, MA, USA) was performed on all individuals. A technician obtained scans of the lumbar spine (L1–L4) and proximal femur (neck and total hip) in the postero-anterior projection. The individuals for whom it was impossible to assess at least three vertebral bodies were excluded from the analysis. Individuals with fractured lumbar vertebrae and vertebrae exhibiting major degenerative changes on radiography were excluded from the analysis to avoid artefacts in the measurement of the BMD. To calculate the T and Z score at the lumbar spine and proximal femur, we used the reference data supplied by the manufacturer. An experienced radiologist blinded to study information read all DEXA scans. Results are expressed as BMD (g/cm2) obtained by dividing the mineral content in the region of interest (ROI) by the area of the region. Each individual was classified on the basis of T score as being either normal (T score>–1.0), osteopenic (T score between –1.0 and –2.5) or osteoporotic (T score≤ –2.5), following the World Health Organization diagnostic criteria [27].

### Statistical analysis

Continuous data were analyzed with Student's *t* test whereas the nonparametric Mann-Whitney test was applied for values not normally distributed. Pearson’s correlation coefficient was used for correlations. One-way ANOVA analysis was used to compare the three study groups. A linear regression model was tested to evaluate the association between T-cell lymphocytes phenotype, OPG, T-score and c-IMT. To explore the factors independently associated with BMD, a multivariable logistic regression was performed. ROC (Receiver Operating Characteristic) curve analysis was performed by PRISM version 5 for Windows (Grafpad software macKiev). Data were expressed as median (range) or mean±standard deviation (SD), as appropriate. A *p* value of <0.05 was considered statistically significant. Statistical analyses were performed using STATA (version 9) software (STATA Corp. LP, College Station, TX).

## Results

### Study population

Out of 94 HIV-infected subjects with a low risk of cardiovascular diseases (Framingham score <10%), 72 (76.5%) were males and 22 (23.5%) females with a mean age of 47.4±11.4 years. 52.1% were not smokers and mean BMI was 20.7±2.4 kg/m^2^. Median nadir and current T lymphocytes CD4^+^ cells count was 195.5 cell/mmc (range 4–1318) and 643.5 cell/mmc (range 159–1705), respectively. Out of 94 HIV-infected subjects on ART, 62.7% were on protease inhibitors and 37.3% on non-nucleoside reverse-transcriptase inhibitors based regimen. All the patients were cytomegalovirus (CMV) positive. The mean plasma concentration of vitamin D was 36.5±4.3 ng/dl, serum creatinine was 0.94±0.21 mg/dl, total cholesterol was 188.3±47.4 mg/dl, HDL cholesterol was equal to 48±14.6 mg/dl, LDL cholesterol was equal to 114±46.3 mg/dl and triglycerides were equal to 145.3±80.9 mg/dl. Mean SBP was 120.3±12.5 mmHg and mean DBP was 80.2±10.6 mmHg. None of the subjects was receiving lipid-lowering therapy. Clinical characteristics are summarized in Table 1.

**Table 1:** Clinical Characteristics.

	HIV+ (n = 94)	HIV- (n = 41)	*p-value*
Age	47.4±11.4	45±10	*p = 0*.*338*
Sex	72 M, 22 F	30 M, 11 F	*p = 0*.*645*
Smoke status (n, %)			
no	49 (52.1%)	24 (58.5%)	*p = 0*.*621*
yes	45 (47.9%)	17 (41.5%)	*p = 0*.*539*
CD4+ (mmc)	685.8±395.5	705±234	*p = 0*.*685*
CD4+ %	27.3±7.6	34±8.3	*p = 0*.*753*
CD4+ (mmc) nadir	242.3±227.6	-	n.a.
CD4+ % nadir	14.7±9.6	-	n.a.
HIV-RNA zenith (cp/ml)	297218.1±114812.4	-	n.a.
HIV-RNA (cp/ml)	<37	<37	n.a.
ART (n, %)	94/94 (100%)	-	n.a.
PI based regimen	59/94 (62.7%)		
NNRTI based regimen	35/94 (37.3%)		
CMV serostatus (n, %)	94/94 (100%)	41/41 (100%)	n.a.
Lipid-lowering therapy	0 (0%)	0 (%)	n.a
SBP (mmHg)	120.3 ± 12.5	113.8 ± 19.4	*p = 0*.*465*
DBP (mmHg)	80.2 ± 10.6	79.5± 13.2	*p = 0*.*285*
Triglycerides (mg/dl)	145.3±80.9	110.3±39	*p = 0*.*721*
Total Colestherol (mg/dl)	188.3±47.4	134.3±48.4	*p = 0*.*775*
Colestherol HDL (mg/dl)	48±14.6	50.3±15.6	*p = 0*.*386*
Colestherol LDL (mg/dl)	114±46.3	113.2±26.2	*p = 0*.*291*
BMI (kg/m2)	21±2.2	20±1.9	*p = 0*.*634*
Framingham score (%)	7.1±2.8	4.2 ± 2.6	*p = 0*.*454*

Values are presented as mean (M) ± standard deviation (SD); ART: AntiRetroviral Therapy; PI: Protease Inhibitor; NNRTI: Non-nucleoside reverse-transcriptase inhibitor; CMV: Cytomegalovirus; SBP: Systolic Blood Pressure; DBP: Diastolic Blood Pressure; HDL: High Density Lipoprotein; LDL: Low Density Lipoprotein. BMI: Body Mass Index.

### Carotid-intima media thickness (c-IMT) and bone damage (DEXA) assessment

**Fig 1:**
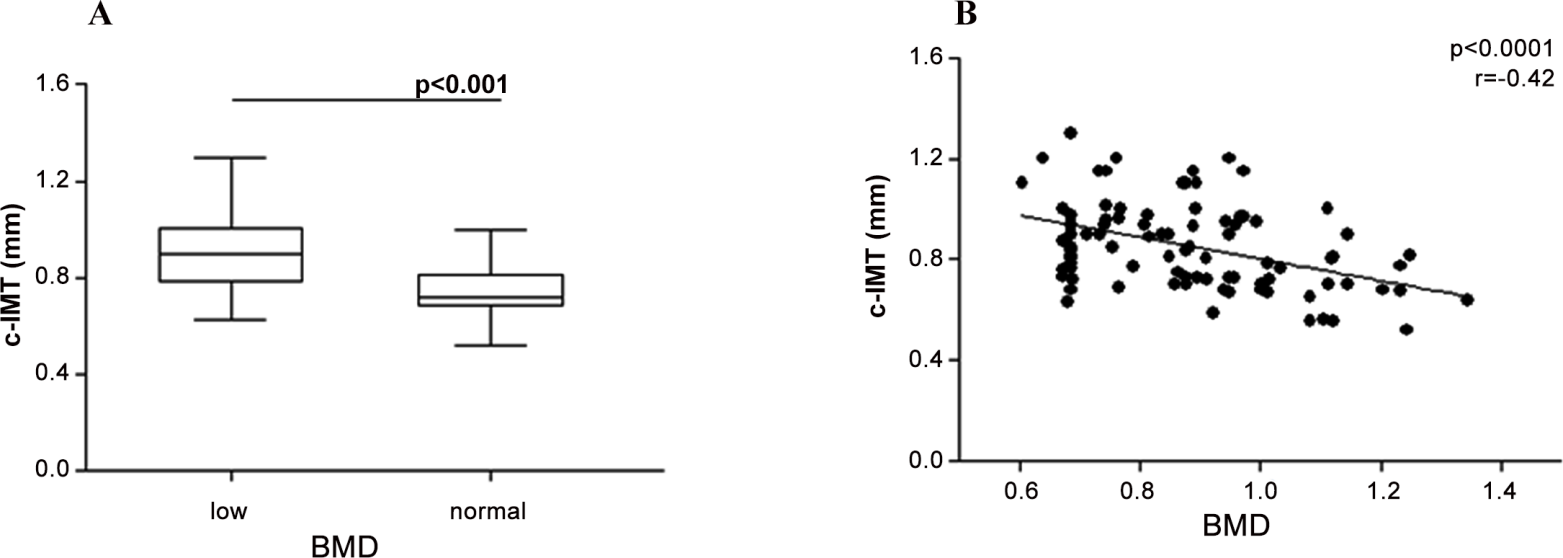
c-IMT and BMD in HIV infected subjects. **A. A** higher c-IMT in HIV positive patients with a low BMD than in patients with a normal BMD was found. Horizontal bars represent the median. The upper and lower whisker indicate the third quartile +1.5 [Inter Quartile Range (IQR)] and first quartile -1.5(IQR). **B. A** linear regression analysis showed a negative correlation between c-IMT and BMD.

Overall, 48/94 (51.1%) had two comorbidities, 16/94 (17%) had one comorbidity and 30/94 (31.9%) no comorbidity. c-IMT was higher in the HIV positive group than in the control group (mean±SD: 0.85±0.17 *vs* 0.48±0.07 mm, p<0.0001). Among HIV positive patients, 52/94 (55.3%) demonstrated a normal c-IMT whereas 42/94 (44.7%) had a pathological c-IMT. The mean BMD value was 0,88±0.17 g/cm2, accordingly 42/94 (44.6%) had osteopenia/osteoporosis whereas 52/94 (55.4%) had normal BMD. 7/42 (16.6%) had osteoporosis and 35/42 (83.4%) osteopenia. Univariate analysis showed a positive correlation between c-IMT and age (p<0.001). A negative correlation between the c-IMT and nadir T cells CD4^+^ (p = 0.008) emerged. Indeed, the HIV positive subjects with pathological c-IMT value had lower percentages of nadir T lymphocytes CD4^+^ compared with those with normal c-IMT value (p = 0.032). Moreover, univariate analysis showed a negative correlation between BMD and age (p<0.001), nadir and current T cells CD4 (p = 0.034 and p = 0.008, respectively), OPG (p = 0.037). HIV positive subjects with concomitant bone and endovascular damage (n = 48) had an increased age (p<0.001) than the other two groups. HIV positive patients with osteopenia/osteoporosis showed a higher c-IMT than patients with a normal BMD (M±SD: 0.910±0.171 mm *vs* 0.789±0.157 mm; p<0.001) (Fig 1A). In univariate linear regression analysis, a negative correlation between c.IMT and BMD (p<0.0001) was found (Fig 1B).

### T cells phenotype and osteoprotegerin: correlation with bone and endovascular damage

When compared to HIV-negative patients, HIV-positive subjects exhibited higher levels of T lymphocytes CD4^+^ expressing HLA-DR^+^CD38^+^ (median values 1.6% *vs* 0.4%, p<0.0001) and CD8^+^ HLA-DR^+^CD38^+^ (median values 2.1% *vs* 0.8%, p<0.0001). HIV-positive subjects exhibited a higher level of T lymphocytes CD4^+^ and CD8^+^ CD28^-^CD57^+^ (median values 2.08% *vs* 1%, p = 0.01, median values 7.44% *vs* 0.65%, p<0.0001, respectively) compared with HIV-negative patients. In univariate linear regression analysis, a positive correlation between CD8^+^ HLA-DR^+^CD38^+^ and CD8^+^ CD28^-^CD57^+^ (p = 0.002) was observed. In addition, there was an inverse correlation between the nadir T cells CD4^+^and T cells CD4^+^ HLA-DR^+^CD38^+^ (p = 0.007). Despite not reaching statistical significance, a similar trend was observed for nadir T cells CD4^+^ and immuneactivated CD8^+^ and immunosenescent, CD4^+^and CD8^+^ cells. There was a positive linear regression between c-IMT and T lymphocytes CD8^+^ CD28^-^CD57^+^ (p<0.001) and an inverse correlation between BMD and T lymphocytes CD8^+^ CD28^-^CD57^+^ (p = 0.008).

**Fig 2:**
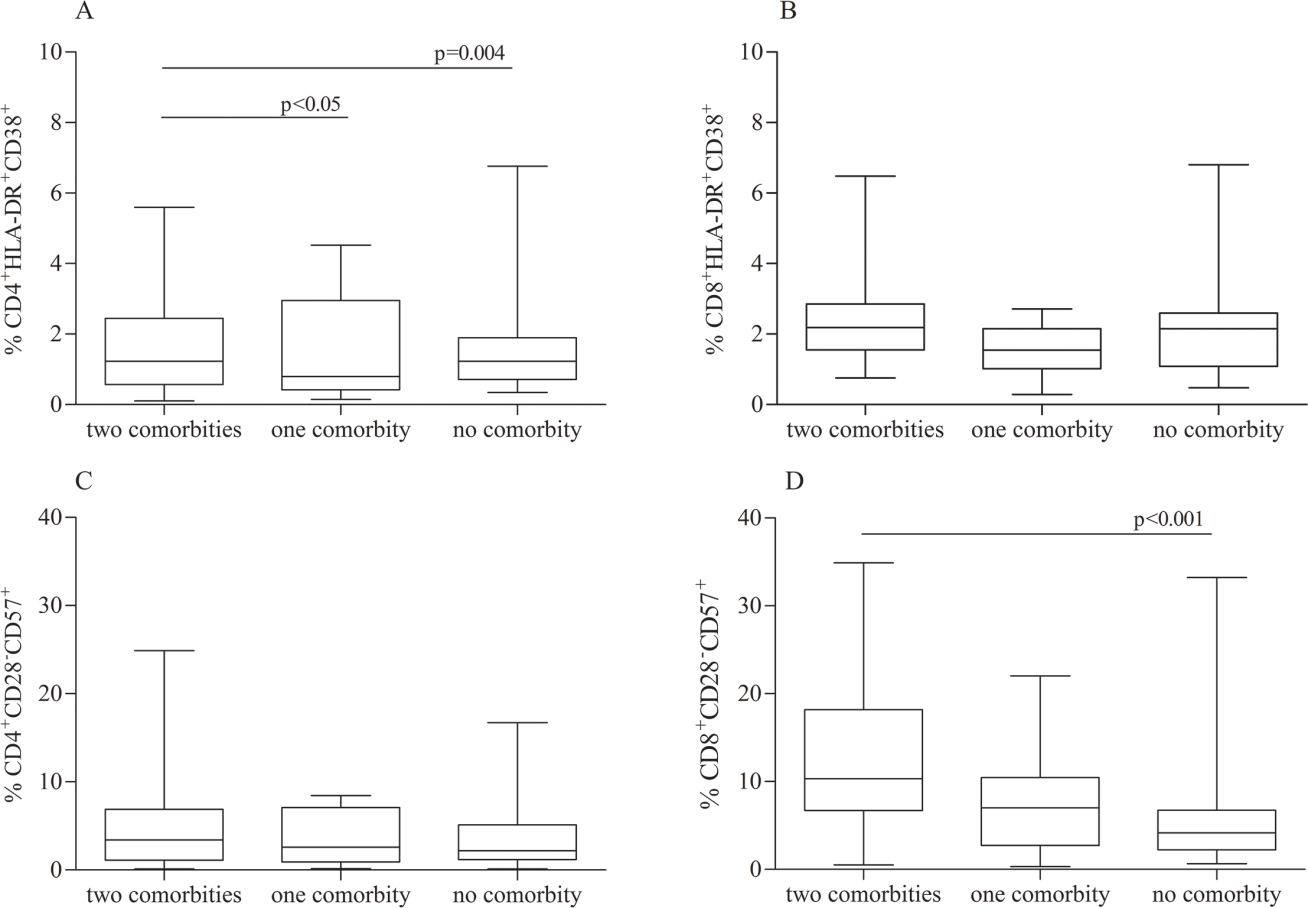
T cells phenotype in HIV infected subjects. CD4^+^ and CD8^+^ immune activation (A, B) and immunesenescence (C, D) in HIV-infected subjects according to the presence of bone and vascular diseases: presence of two comorbidties (bone and endovascular damage), only one comorbidity (bone or endovascular damage), no comorbidity. Values are expressed as a percentage. The horizontal bars represent the median. The upper and lower whisker indicate the third quartile +1.5 [Inter Quartile Range (IQR)] and first quartile -1.5(IQR).

The levels of immune activation of T cells CD4^+^ were found to be higher in HIV-positive subjects with concomitant bone and endovascular damage compared to the other groups (median CD4^+^ HLA-DR^+^CD38^+^: 2.2% Group A *vs* 0.79% Group B *vs* 1.23% Group C, p = 0.004) whereas the same trend was found for T cells CD8^+^ HLA-DR^+^CD38^+^ without reaching statistical significance (median CD8^+^ HLA-DR^+^CD38^+^: 2.2% Group A *vs* 1.78% Group B *vs* 2.1% Group C, p = 0.087) (Fig 2A and 2B). Similarly, levels of immunosenescent lymphocyte T CD8^+^ cells were found to be higher in HIV-positive subjects with concomitant bone and endovascular damage compared to the other groups (median CD8^+^ CD28^-^CD57^+^: 10.3% Group A *vs* 7.9% Group B *vs* 4.1 Group C%, p<0.001) whereas the same trend was found for T cells CD4^+^ CD28^-^CD57^+^without reaching statistical significance (median CD4^+^ CD28^-^CD57^+^: 2.1% Group A *vs* 2% Group B *vs* 2% Group C, p = 0.867, (Fig 2C and 2D).

**Fig 3:**
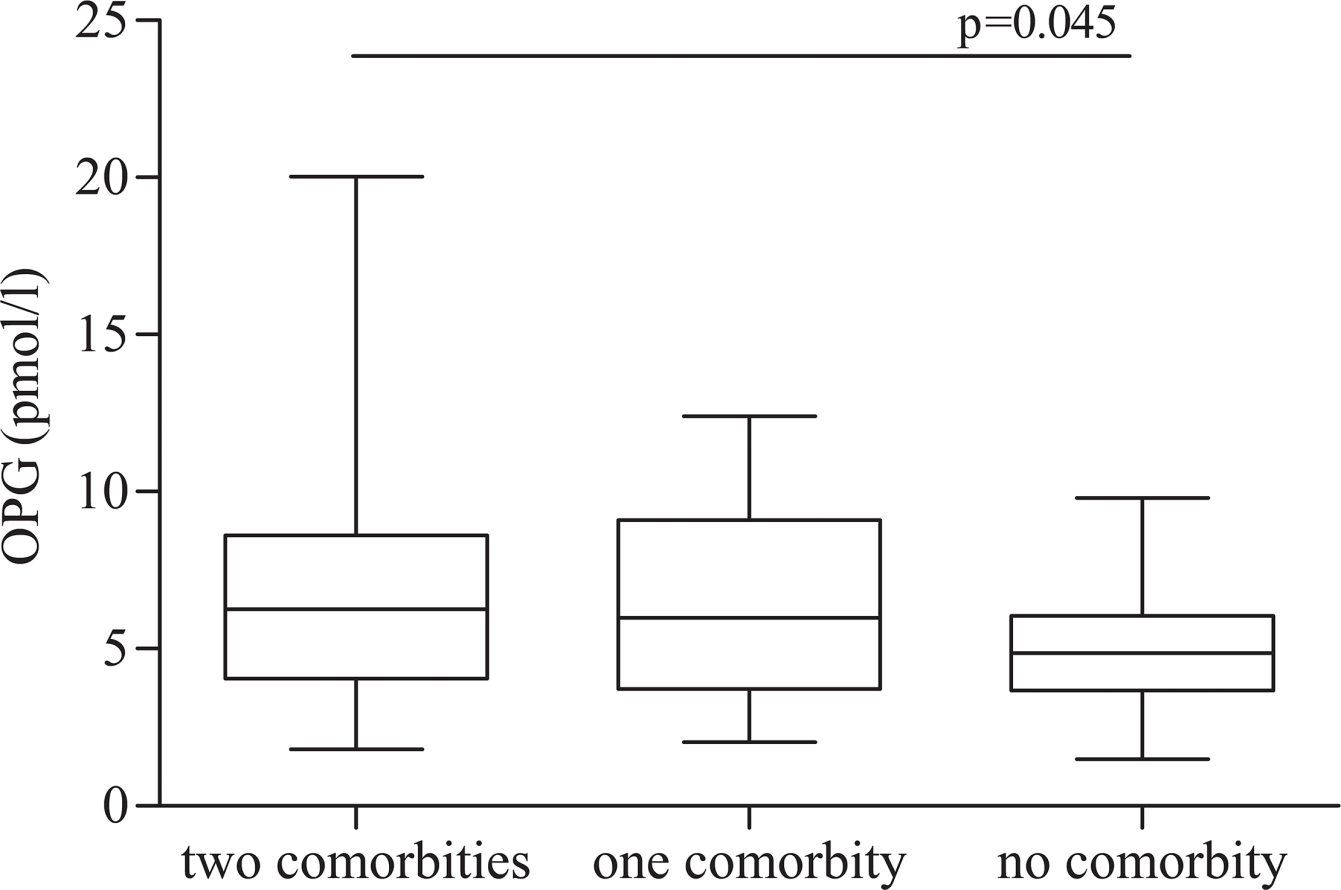
OPG plasma levels in HIV positive subjects. OPG plasma levels (pmol/l) in HIV-infected subjects according to the presence of bone and endovascular damage: presence of two comorbidties (bone and endovascular damage), only one comorbidity (bone or endovascular damage), no comorbidity. The horizontal bars represent median. The upper and lower whisker indicate the third quartile +1.5 [Inter Quartile Range (IQR)] and first quartile -1.5(IQR).

OPG plasma levels were significantly higher in HIV-infected patients than in healthy controls (mean±SD: 6.59±4 pmol/l *vs* 3.5±1.2 pmol/l) (p<0.0001). In the univariate linear regression analysis, there was a positive correlation between plasma levels of OPG and CD8^+^ HLA-DR^+^CD38^+^ (p = 0.008). Moreover, the same trend was observed between OPG and T cells CD4^+^ HLA-DR^+^CD38^+^, T cells CD4^+^ and CD8^+^ CD28^-^CD57^+^, although not statistically significant. OPG plasma levels were found to be higher in HIV-positive subjects with concomitant bone and endovascular damage compared to the other groups (mean±SD: 7.29±4.17 pmol/l Group A *vs* 7.74±5.9 pmol/l Group B *vs* 4.98±1.77 pmol/l Group A, p = 0.045) (Fig 3). Multivariable logistic regression showed that only age (OR = 1.166; confidence interval [CI] = 1.081–1.259; p<0.001) and T lymphocytes CD8^+^ CD28^-^CD57^+^ (OR = 1.097; CI = 1.014–1.186; p = 0.021) were independently associated with the presence of two comorbidities (Table 2).

**Table 2:** Multivariate Analysis.

	OR	95% IC	*p*
Age	1.166	1.081–1.259	**<0.001**
Time of Diagnosis	1.034	0.958–1.116	0.392
Smoke Status	0.657	0.205–2.107	0.480
Nadir CD4^+^	0.997	0.994–1.001	0.100
OPG	0.995	0.867–1.141	0.942
CD4^+^ HLA-DR^+^CD38^+^	1.381	0.920–2.073	0.119
CD4^+^ CD28^-^CD57^+^	1.038	0.896–1.203	0.620
CD8^+^ HLA-DR^+^CD38^+^	0.890	0.733–1.080	0.238
CD8^+^ CD28^-^CD57^+^	1.097	1.014–1.186	**0.021**

Multivariable logistic regression showed that only age and T lymphocytes CD8^+^ CD28^-^CD57^+^ were independently associated with the presence of two comorbidities in HIV positive subjects. OR: Odd Ratio; CI: Confidence Interval; OPG: Osteoprotegerin.

**Fig 4:**
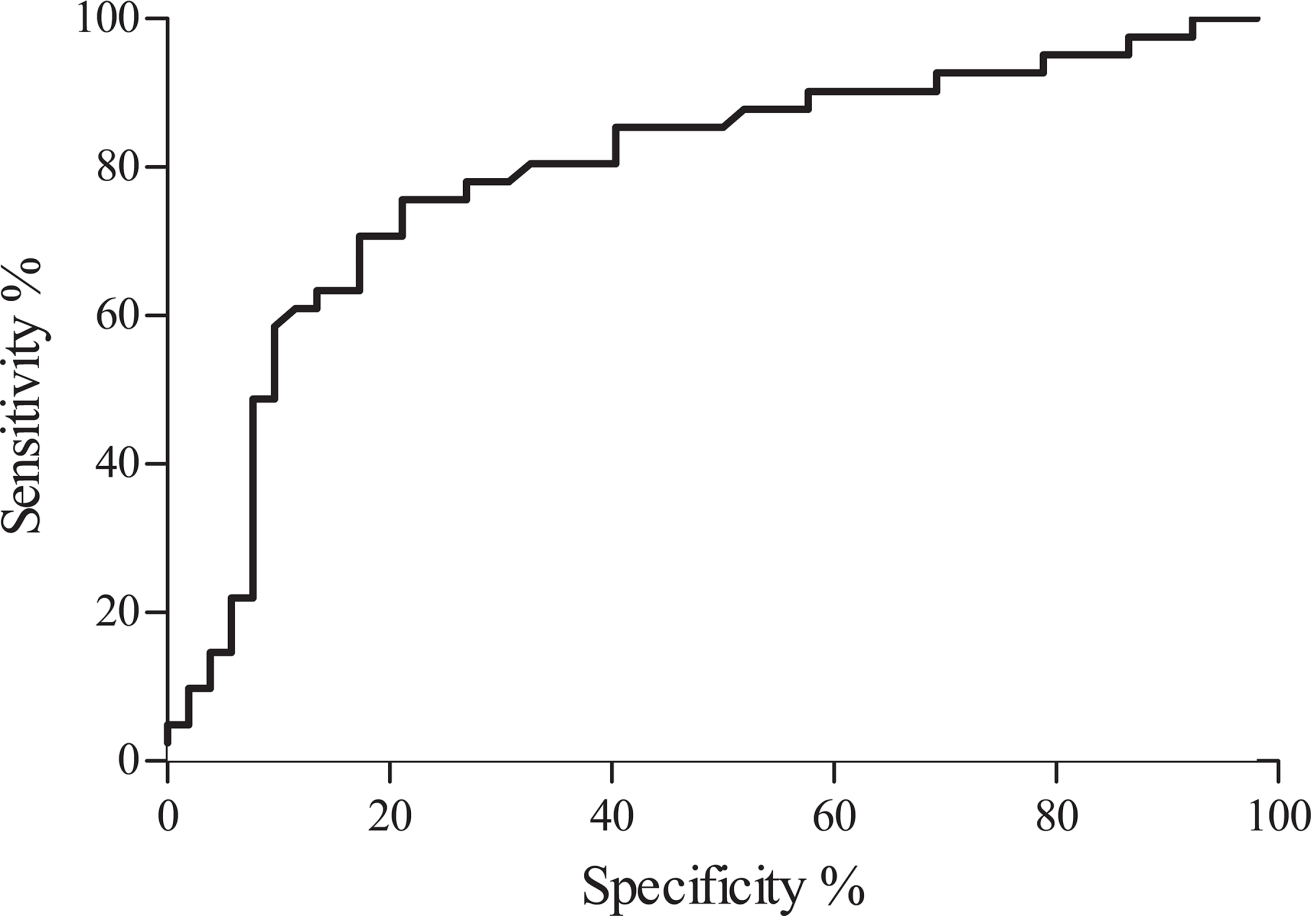
ROC curve analysis. The ROC curve area for CD8^+^CD28^-^CD57^+^ (AUC) was 0.79 and the cut-off >8.87% has a sensitivity of 75,61% (CI: 59.7% to 87.64%), specificity of 78.85% (CI: 65.30% to 88.94%) and a likelihood ratio of 3.57 (p<0.0001).

A ROC curve was performed to establish the cut-off point of T lymphocytes CD8^+^ CD28^-^CD57^+^ that correlated with the presence of two comorbidities. For a CD8^+^ CD28^-^CD57^+^ cut-off >8.87%, the area under the curve (AUC) was 0.79 with a sensitivity of 75,61% (CI: 59.7% to 87.64%), specificity of 78.85% (CI: 65.30% to 88.94%) and a likelihood ratio of 3.57 (p<0.0001) (Fig 4).

## Discussion

Although antiretroviral therapy has changed the length and quality of life of HIV-positive patients, morbidity and mortality due to non-AIDS-related complications, such as osteoporosis and atherosclerosis, were increased, even in the presence of viral suppression [1]. Traditionally, these two conditions have always been considered not related to each other and their coexistence was exclusively an age-associated process [28]. However, recent studies have shown that there is a possible link between the two pathological conditions by demonstrating that in the general population, subjects with osteoporosis are at increased risk of developing cardiovascular disease compared to those with normal bone mass [5,29–30]. Although a recent study showed that there was an increased in CD161-expressing and TNF secreting invariant Natural killer T cells in HIV positive subject with concomitant bone and cardiovascular disease, suggesting that these cells might have a role in the pathogenesis of non infectious co-morbidities in HIV population, little is known on other cellular subsets involvement in HIV positive patients [31].

Thus, in the present study, the relationship between bone and cardiovascular disease and T-cell phenotype in HIV positive subjects on effective antiretroviral therapy (ART) was evaluated.

Carotid-IMT values were found to be higher in HIV positive subjects than in healthy control and, more interestingly, HIV-positive patients with bone disease had higher values of c-IMT than patients with a normal BMD, as shown by the inverse correlation between BMD and c-IMT found at the univariate analysis. Besides the already known risk factors in the general population (age, dyslipidemia, hypertension, smoking status, diabetes, hypovitaminosis D and calcium deficiency), in HIV subjects there are additional and specific elements that might contribute to the cumulative risk of developing cardiovascular and bone damage [32–34]. In fact during HIV infection, a higher activation of T lymphocytes was observed, resulting in the production of several inflammatory cytokines with subsequent state of chronic inflammation, even in the case of an effective ART [35].

In the present study, HIV-positive subjects with concomitant bone and endovascular damage showed a higher state of immune activation and immunosenescence of T cells CD4^+^ and CD8^+^ plasma levels than subjects with one or no comorbidities.

It is known that HIV determines a state of chronic inflammation with activation and progressive aging of the immune system. An important aspect of this premature aging is the immune activation and the consequent immunosenescence that causes a thymic involution, a reduced circulating naive T-cells and an increased number of T cells CD28^-^ [8].

In this study, the multivariable logistic regression showed that only age and T lymphocytes CD8^+^ CD28^-^CD57^+^ were independently associated with the presence of two comorbidities; in particular, the ROC curve revealed that a cut-off>8.87% of T lymphocytes CD8^+^ CD28^-^CD57^+^ correlated with the presence of concomitant bone and cardiovascular disease, suggesting that the immune exhaustion during HIV infection might exert a prominent role in the development of these conditions.

In the setting of the immune dysregulation present in HIV subjects, the role of traditional pro-inflammatory cytokines such as IL-1, IL-6 and TNF-α has been widely demonstrated [15]. Recently, the RANK/RANKL/OPG axis has emerged as a possible link between osteoporosis and cardiovascular disease by playing a crucial role in the interaction between the immune system and the development of cardiovascular and bone disease both in healthy and HIV positive subjects [22–23, 36].

This hypothesis was confirmed by the results of the present study, in which a higher plasma concentration of OPG in HIV positive subjects than healthy control and a positive correlation with T lymphocytes CD8^+^ immune activated were found. Moreover, among HIV positive subjects, OPG was higher in those who had concomitant bone and cardiovascular damage than in those who had one or not comorbidity.

In fact, it has been shown in *in-vitro* studies that T lymphocytes influence the basal production of OPG, especially in conditions of inflammatory states, where the activated T cells become a significant additional RANKL source [37–38]. In these conditions, the high levels of RANKL might be due to a greater synthesis of RANKL and/or to an insufficient increase of OPG synthesis leading to an elevated RANKL/OPG ratio, which is associated with bone resorption and atherogenesis. On the other hand, *in-vivo* studies demonstrated that OPG plasma levels were significantly higher in HIV-positive subjects than in healthy controls [36]. These data suggest that HIV infection itself, together with ART, could act on T lymphocytes, osteoblasts and stromal cells by increasing OPG synthesis and resulting in a compensatory mechanism to counterbalance the expression of RANKL by these activated cells. The imbalance of the RANKL/OPG *ratio* was associated with an alteration in the production of osteoblasts/osteoclasts at the onset of bone disease in HIV positive patients and in the development of cardiovascular disease, although there are still few and conflicting data about it. In particular, it seems that OPG *in-vitro* protects vascular calcification whereas *in-vivo* higher OPG plasma levels are significantly associated with endothelial dysfunction and the development of coronary heart disease [36, 39–40]. In a previous study conducted by our group, high OPG plasma levels were found in HIV patients with low cardiovascular risk but with organ damage, suggesting that OPG plays a role in the early stages of the atherosclerotic process [41].

The mechanism throughout OPG might contribute to cardiovascular disease relies on endothelial dysfunction by blocking RANKL, which is known to be able to activate protective pathways in endothelial cells such as nitric oxide synthase. In addition, OPG would increase the adhesion and migration of inflammatory cells across the endothelium, the activity of metalloproteinases, the inhibition of apoptosis of inflammatory cells, smooth muscle cells and endothelial cells with increased plaque instability [42–43].

The scenario of multiple comorbidities during HIV infection is complicated: virus, ART, immune activation and immunosenescence with pro-inflammatory cytokines play a predominant role [44–45]. Several hypotheses have been advanced to explain the possible relationship between bone and vascular disease as they recognize both risk factors and common pathophysiological mechanisms. Despite the fact that traditional factors should always considered in the pathogenesis of non-AIDS related comorbidities in HIV-positive subjects, the perturbation of the OPG/RANK/RANKL pathway, together with inflammation and immunesenescence, could affect bone turnover and the vascular system, even in the case of viral suppression.

## Conclusion

In the presence of a complex but still not fully understood interaction between HIV, immune system and RANKL/OPG axis, OPG plasma levels together with the evaluation of immunosenescent CD8^+^ T cells might represent useful and non-invasive parameters in order to identify early organ damage in asymptomatic HIV-positive subjects.
